# A Novel Method of Determining Portal Systemic Shunting using Biodegradable ^99^TC^m^ Labelled Albumin Microspheres

**DOI:** 10.1155/1995/31761

**Published:** 1995

**Authors:** J. Yates, D. M. Nott, P. J. Maltby, D. Billington, J. N. Baxter, R. Shields, S. A. Jenkins

**Affiliations:** 1 Departments of Surgery and Nuclear Medicine Royal Liverpool Hospital Prescot Street Liverpool L7 8XP UK; 2 School of Natural Sciences John Moores University Byrom Street Liverpool L3 3AF UK; 3 Department of Surgery Royal Liverpool Hosphital Liverpool L7 8XP UK

## Abstract

Portal systemic shunting (PSS) and portal pressure were measured in control rats and in animals with portal hypertension induced by partial portal vein ligation (PPVL). The portal pressure in rats with partial portal vein ligation (13.4 ± 0.5 mm.Hg.) was significantly higher (*p* < 0.005) than in the control group (9.6 ± 0.6 mm.Hg.). Portal systemic shunting measured by consecutive injections of radiolabelled methylene diphosphonate (MDP), a non-diffusable marker and albumin microspheres directly into the splenic pulp was significantly increased (*P* < 0.005) in the portal hypertensive animals (30.8 ± 2.5%) compared to sham operated rats (2.6 ± 1.5%). Similarly, in portal hypertensive rats portal systemic shunting measured by intrasplenic injections of radiolabelled cobalt microspheres (37.1 ± 3.9%) was significantly greater (*p* < 0.005) than in control animals. There was a good correlation and agreement (*r* = 00.97) between the two methods of measuring portal systemic shunting. However because the ^99^Tc^m^-albumin microspheres are biodegradable the method allows portal systemic shunting to be measured in man. Furthermore since the computer adjusts the baseline to zero after each determination of portal systemic shunting the methodology allows repeated measurements to be made.


*HPB Surgery*, 1995, Vol. 8, pp. 223–229
Reprints available directly from the publisher
Photocopying permitted by license only

© 1995 OPA (Overseas Publishers Association)
Amsterdam B.V. Published in The Netherlands
by Harwood Academic Publishers GmbH
Printed in Malaysia

### A Novel Method of Determining Portal Systemic Shunting using Biodegradable $^{99}Tc^{m}$ Labelled Albumin Microspheres

J. YATES, D. M. NOTT, P. J. MALTBY*, D. BILLINGTON**, J. N. BAXTER, R. SHIELDS and S. A. JENKINS

*Departments of Surgery and Nuclear Medicine, Royal Liverpool Hospital Prescot Street, Liverpool L7 8XP, U.K.
**School of Natural Sciences, John Moores University, Byrom Street, Liverpool L3 3AF, U.K.

*(Received October 28, 1993)*

Portal systemic shunting (PSS) and portal pressure were measured in control rats and in animals with portal hypertension induced by partial portal vein ligation (PPVL). The portal pressure in rats with partial portal vein ligation (13.4 ± 0.5 mm.Hg.) was significantly higher ($p < 0.005$) than in the control group (9.6 ± 0.6 mm.Hg.). Portal systemic shunting measured by consecutive injections of radiolabelled methylene diphosphonate (MDP), a non-diffusable marker and albumin microspheres directly into the splenic pulp was significantly increased ($P < 0.005$) in the portal hypertensive animals (30.8 ± 2.5%) compared to sham operated rats (2.6 ± 1.5%). Similarly, in portal hypertensive rats portal systemic shunting measured by intrasplenic injections of radiolabelled cobalt microspheres (37.1 ± 3.9%) was significantly greater ($p < 0.005$) than in control animals. There was a good correlation and agreement ($r = 00.97$) between the two methods of measuring portal systemic shunting. However because the $^{99}Tc^{m}$-albumin microspheres are biodegradable the method allows portal systemic shunting to be measured in man. Furthermore since the computer adjusts the baseline to zero after each determination of portal systemic shunting the methodology allows repeated measurements to be made.

KEY WORDS: Portal hypertension portal systemic shunting rats microspheres

### INTRODUCTION

Bleeding from gastro-oesophageal varices is a serious and often fatal complication of portal hypertension in chronic liver disease. Variceal haemorrhage rarely occurs unless the portal pressure exceeds 12 mm Hg[1]. However, no relationship has been demonstrated between the magnitude of portal pressure and the occurrence of variceal bleeding[2]. A possible explanation for this observation is the relationship between portal pressure and the blood flow through the collateral circulation including the gastro-oesophageal varices. Thus, although a good correlation has been demonstrated between azygos blood flow (an indirect measurement of collateral blood flow) and portal pressure in man, the relationship is exponential. Thus, when the portal pressure is only mildly elevated above normal in portal hypertensive patients, further relatively large increases in pressure result in only modest elevations in azygos blood flow[3]. Conversely when the portal pressure is markedly elevated, further small increases in pressure result in large elevations in azygos blood flow[3]. Furthermore, recent studies have indicated that vasoactive drugs have a differential action on portal pressure and azygos blood flow[4,5]. Measurement of collateral blood flow therefore gives information over and above that available from measurements of portal pressure alone both with respect to the pathophysiology of variceal bleeding and the effects of vasoactive drugs used to control haemorrhage and long-term management of portal hypertension.

*Address for correspondence:* S. A. Jenkins, Department of Surgery, Royal Liverpool Hosphital, Liverpool, L7 8XP U.K.

Currently measurement of azygos blood flow is the method of choice for measuring collateral blood flow in man. However, the technique is relatively difficult, expensive and confined to centres that have particular expertise in determining azygos blood flow. Furthermore, the azygos system drains blood not only from the collateral circulation, but also from the posterior costal, mediastinal and bronchial veins. It generally assumed that alterations in azygos blood flow in portal hypertensive patients reflect changes in collateral blood flow and that the proportion of blood draining into the system from the other veins is relatively constant. However, this may not be the case if cardiac output and regional blood flow are both altered, which is often the case in portal hypertension. Therefore, there is clearly a need for a simple method of measuring collateral blood flow *per se*. In portal hypertensive animals collateral blood flow can be measured directly by intrasplenic injection of non-biodegradable radiolabelled microspheres[6]. This technique is obviously not applicable to patients. Furthermore, even in experimental animals the microsphere method of determining collateral blood flow has limitations. Thus, the number of consecutive measurements of collateral blood flow is limited by the need to select microspheres labelled with isotopes of widely varying energies to facilitate subsequent counting. Although numerous attempts have been made to determine extrahepatic PSS scintigraphically after administration of biodegradable radiolabelled aggregates (MAA) or microspheres of human serum albumin, there are a number of methodological difficulties in the techniques hitherto described. Thus, in the majority of studies a radiolabelled reference marker extracted by the Kuppfer cells is used in conjunction with either human albumin macroaggregates (MAA) or microspheres. However, implicit in the calculation of a shunt index is the assumption that the reference marker is almost completely extracted from the blood by the Kuppfer cells on a single passage through the liver. This is clearly not the case in liver disease since Kuppfer cell activity is compromised, often severely, in patients with advanced disease. Furthermore, repeated estimations of PSS are not possible since the Kuppfer cells become saturated with reference marker. We describe here a method for measuring collateral blood flow using consecutive injection of $^{99}Tc^{m}$ methylene diphosphonate and $^{99}Tc^{m}$-albumin microspheres directly into the splenic pulp. Since the computer software recalibrates the baseline after each injection of radioisotope, the technique allows multiple measurements of collateral blood flow to be made.

### METHODS

### *Radiolabelling of microspheres*

Reaction vials contained 2.5 mg Human Serum Albumin microspheres (Rotop Akademie der Wissenschaften, Germany) 0.1 mg $SnCl_2 2H_2O$ and 0.6 mg Tween 80. (pH 4.7). Approximately $3–5 \times 10^5$ microspheres with a mean diameter of 20 $\mu$ (range 10–30 $\mu$) were present in 2.5 mg of albumin microspheres. The vials were reconstituted with 300 MBq of $^{99m}TcO_4$ in a volume of 3 ml isotonic saline. The labelling yield was in the order of 99% as ascertained by paper chromatography using methanol: water (85:15) as the solvent.

Carbonised microspheres (NEN-Trac, Dupont), 15.5 $u \pm 0.1$ $\mu$ diameter labelled with $^{57}$Cobalt ($^{57}$Co), $^{51}$Chromium ($^{51}$Cr) and $^{46}$Scandium ($^{46}$Sc) were suspended in 0.9% saline and 0.01% Tween 80. Vials contained $3.04 \times 10^5$ microspheres per mg with 41.3 mg per vial.

### *Experimental design*

Male Wistar rats, 250–350g body weight, were randomly assigned to one of two groups, each containing twenty-two animals. One group underwent partial portal vein ligation[7] to induce portal hypertension and the development of a substantial collateral circulation. Briefly, (after an overnight fast) the rats were anaesthetised by an intramuscular injection of Immobilon (Etorphine/Methorimeprazine). The abdomen was opened through a midline incision and the portal vein identified and mobilised at a point cranial to its junction with the pancreaticoduodenal vein. A single 5/0 silk ligature was passed around the portal vein and tied so as to include a 20G blunt needle. The needle was withdrawn and the vessel allowed to re-expand, resulting in an approximately uniform stenosis of the portal vein (0.9 mm.) in all animals. The abdomen was closed in a single layer using 3/0 silk sutures and anaesthesia reversed by an intramuscular injection of the etorphine antagonist Revivon (Diprenorphine).

The second group of rats underwent a sham operation in which the abdomen was opened, the portal vein mobilised and the laparotomy closed in single layer.

Hepatic Haemodynamics were determined not less than two weeks and not more than four weeks after partial portal vein ligation or sham operation to allow the development of varying degrees of collateral circulation in the portal hypertensive rats.

At the time of investigation all animals were anaesthetised by intraperitoneal injection of 6 mg/Kg Pentobarbitone Sodium (Sagatal, May and Baker Ltd).

Through a midline incision the portal vein was cannulated via a small branch of the superior mesenteric vein, thus minimising any effect of the procedure on portal venous inflow. The cannula was connected via an isolated pressure transducer (Medex Ltd.) and amplifier to a two channel chart recorder (Lectromed UK Ltd.). The abdominal incision was then closed to minimise heat and evaporative loss. The spleen was exposed through a separate, small, left lateral abdominal incision. The rats were placed under a 50 mm × 50 mm straight bore sodium iodide scintillation detector positioned over the clavicular area to include the upper pole of the lungs and shielded from the liver by a lead shield. The detector was connected through a single channel analyser to a gamma counter and the count rate transferred continuously to a microcomputer (Spherex monitoring system, Pharmacia, Sweden).

The increase in the count rate in the lung field of interest was calculated as the difference between the plateau level after each injection of the radionuclide and the baseline level. The baseline was determined from the mean of the count rate during the thirty seconds immediately before the start of each injection. The plateau level was calculated as the mean count rate during sixty seconds, (the average counts per second for six consecutive ten second periods during the stable phase in each measurement) within a period of three minutes from each injection. The count rate was corrected for the dose of radionuclide in each injection to obtain comparable values of the count rate per unit of radioactivity injected. The decay of $^{99m}$Tc (half-life = 6 hours) and the 'deadtime'of the detector were corrected for by the computer software to obtain a linear response up to a miximum count rate of 65,000 c.p.s.

A reference dose of 15 μl $^{99m}$Tc-methylene diphosphonate (MDP, 100 MBq/ml) was injected directly into the splenic pulp using a high pressure liquid chromatography (HPLC) syringe. MDP is a low molecular weight non-diffusable marker which is not retained within the liver[8] and consequently all the radioactivity injected passes directly to the lung field of interest. Therefore the increase in count rate after the injection of the MDP was calculated as the difference between the plateau level obtained after its injection and the baseline level and was regarded as the 100% passing fraction (Figure 1). The injection of MDP was followed three minutes later by an intrasplenic injection of the same volume of $^{99m}$Tc labelled human serum albumin microspheres. In portal hypertensive rats the proportion of albumin microspheres passing to the lungs is proportional to the degree of extrahepatic shunting and was calculated as a percentage of the 100% passing fraction (Figure 1). In order to establish that the administration of the albumin microspheres had not caused any adverse haemodynamic changes in the splanchnic circulation, a second MDP injection was administered three minutes after the albumin microspheres. If the two MDP curves were within 10% of each other, it was assumed that no haemodynamic alterations had resulted from the injection of the albumin microspheres. If the two MDP curves were not within these limits the animal was excluded from the study.

After completion of the albumin microsphere method determinations of portal systemic shunting were repeated using $^{57}$Co microspheres[6]. In brief, 40 μl of $^{57}$Co microspheres were administered by intrasplenic injection, the animals left for twenty minutes, killed, the liver and lungs excised, weighed and placed in gamma counting tubes. The radioactivity in the liver and lungs was counted in a Canberra Packard Cobra gamma counter seven days later, when all the $^{99m}$Tc had decayed. PSS was calculated using the formula:-

$$\%PSS = \frac{\text{net lung activity} \times 100}{\text{net liver} + \text{lung activity}}$$

*Repeated measurements of PSS*

PSS was determined in 10 portal hypertensive rats by consecutive intrasplenic injections of MDP and $^{99m}$Tc-albumin microspheres repetitively at 10 min. intervals over a period of 50 min. (6 determinations in each animal).

*Statistical analysis*

Paired Student's t-tests were used to compare the degree of portal systemic shunting determined by the two methods in the same animals. An analysis of variance was used for the comparison of portal pressure and portal systemic shunting between partial portal vein ligated animals and corresponding sham operated animals. The correlation between the two methods of measuring portal systemic shunting was determined using Pearson's correlation coefficient. Agreement between the methods was calculated using the method described by Bland and Altman[9]. Data are presented as mean ± standard error of the mean.

### RESULTS

Following the injection of $^{99m}$Tc-labelled albumin microspheres into the splenic pulp of sham operated

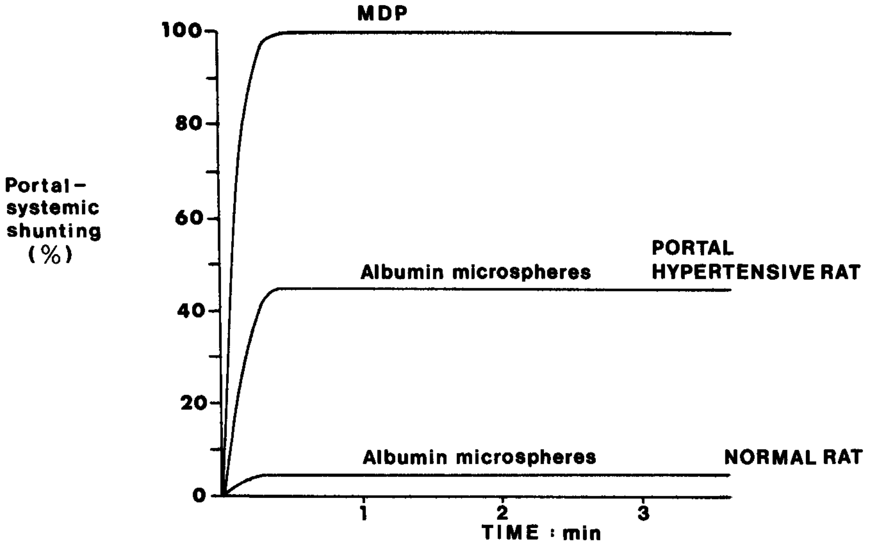

**Figure 1** Diagrammatic representation of the computer printout following intrasplenic injection of $^{99}Tc^m$ MDP and $^{99}Tc^m$ albumin microspheres in normal and portal hypertensive rats. In normal (sham operated) animals almost all of the albumin microspheres are trapped within the liver whereas in portal hypertensive rats a variable fraction passes to the lungs via the collateral circulation.

rats, almost all the radioactivity was trapped within the hepatic capillary bed and little was detected in the lung field of interest. Thus, in normal rats, extrahepatic shunting (2.6 ± 0.7%) was minimal. Similarly, the degree of portal systemic shunting calculated from the lung:liver ratio of radioactivity following injection of $^{57}Co$ microspheres was also very low (2.4 ± 0.6%). There was a good correlation ($r = 0.92$) between the two methods to determine portal systemic shunting in control rats (Figure 2). Partial portal vein ligation resulted in a significant elevation ($p < 0.005$) in portal pressure 13.4 ± 0.5 mm Hg) compared to that (9.6 ± 0.6 mm Hg) in the sham operated group. The degree of PSS measured by both albumin and cobalt microspheres was variable in the portal hypertensive rats and was dependent on the length of time between partial portal vein ligation and the determination of extrahepatic shunting. In general, PSS was greater the longer

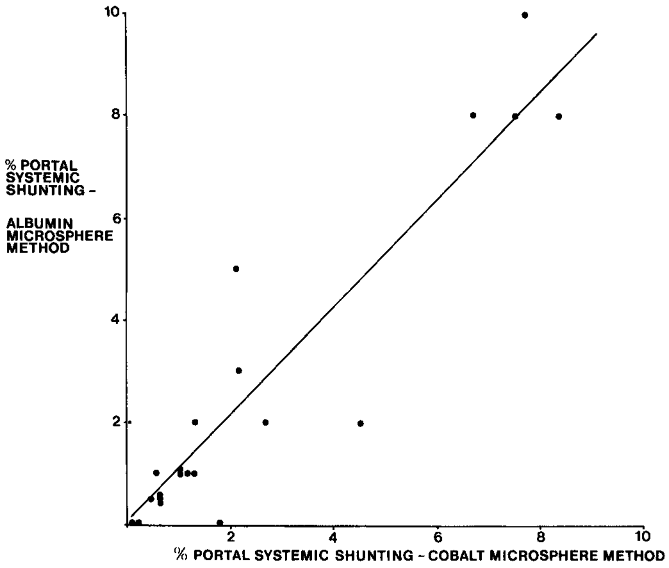

**Figure 2** Correlation of portal systemic shunting determined by the $^{99}Tc^m$-albumin and $^{57}Co$-microsphere methods in sham operated rats ($y = 0.10 + 1.04$; $r = 0.92$).

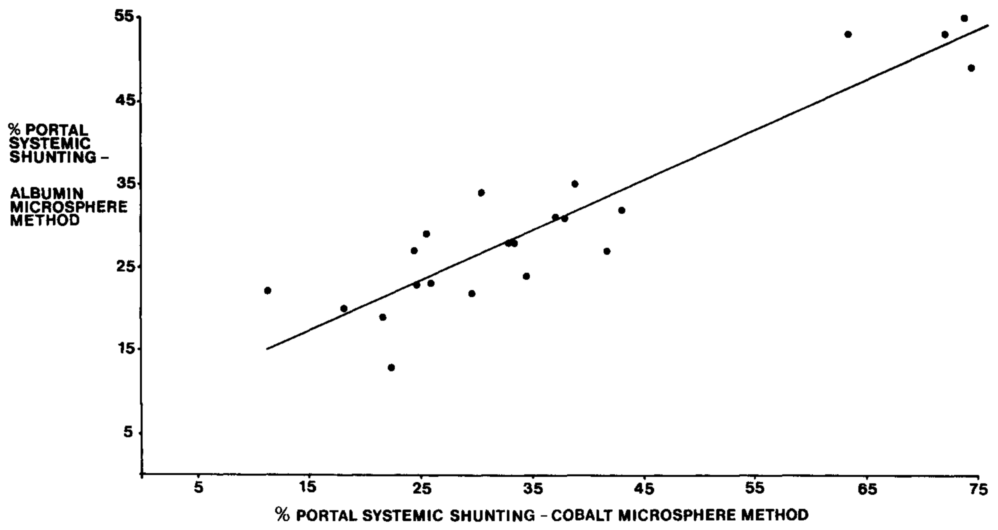

**Figure 3** Correlation between the two microsphere methods of determining portal systemic shunting in rats with partial portal vein ligation, ($y = 8.6 + 0.60x$; $r = 0.93$).

the animals were left after ligation of the portal vein. Nevertheless, portal systemic shunting measured by albumin microspheres (30.8 ± 2.5%) and cobalt microspheres (37.1 ± 3.9%) was significantly increased ($p < 0.001$) compared to corresponding controls.

There was a good correlation ($r = 0.93$) between the albumin and cobalt microsphere methods of measuring portal systemic shunting in rats subjected to partial portal vein ligation (Figure 3). Furthermore combining the results for both control rats and those with PPVL (Figure 4) there was good correlation between the two methods of measuring PSS ($r = 0.97$). The gradient from the regression equation for portal systemic shunting in control rats was 1.04 (Figure 2) and that in portal hypertensive animals was 0.60 (Figure 3). Combining the results from both groups of rats produced a regression equation with a gradient intermediate between these two values (Figure 4). In order to further investigate agreement between the two methods, the combined data of all rats were replotted (Figure 5) by the method described by Bland and Altman[8]. Thus when PSS was low all the data points were clustered near the line of perfect agreement (zero difference). However, as-the degree of PSS increased in the PPVL

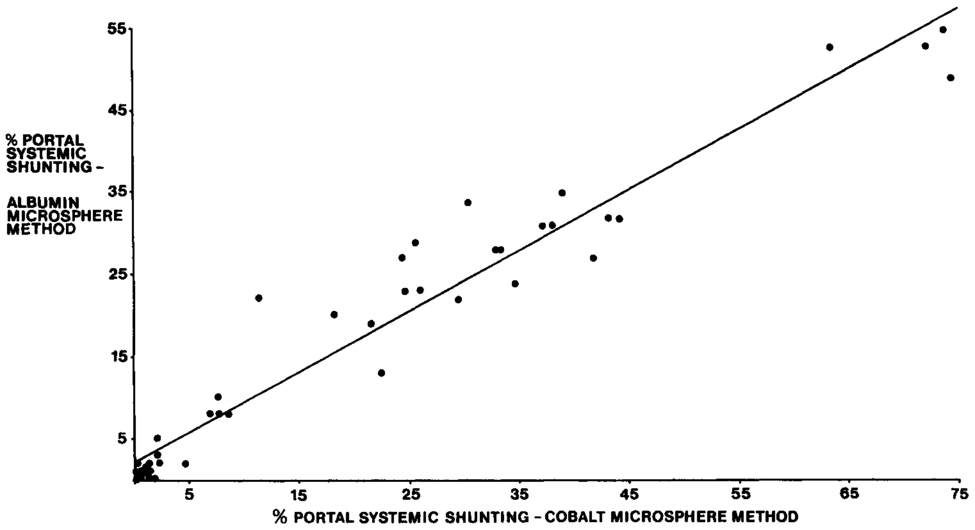

**Figure 4** Correlation between the two microsphere methods of determining portal systemic combining both groups of rats, ($y = 2.10 + 0.74x$; $r = 0.97$).

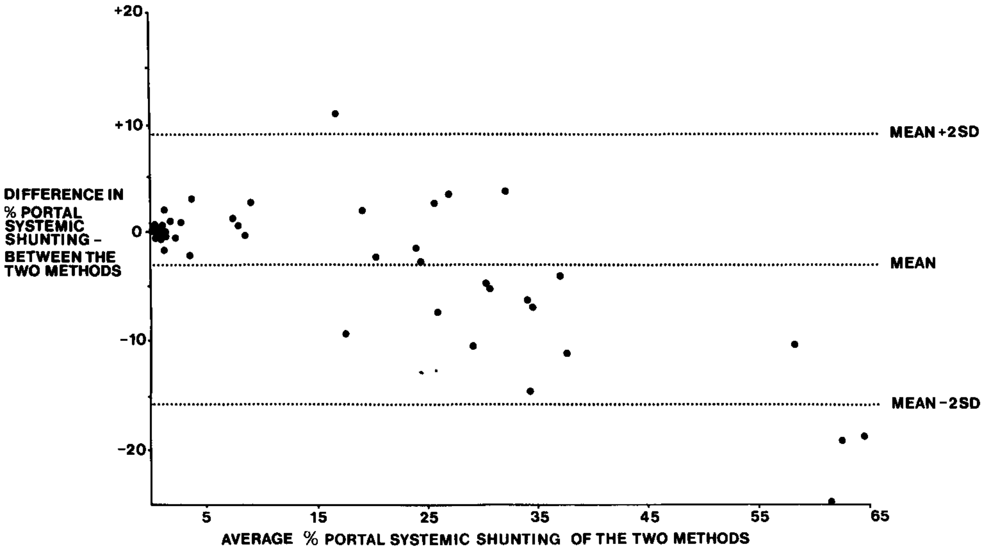

**Figure 5** Relationship of the discrepancy between portal systemic shunting measured by albumin on cobalt microspheres in all rats to the mean PSS by the two techniques.

rats, the data points showed a slight progressive negative bias away from the line of perfect agreement. However, the majority (40/44) of the data points fell within the limits of agreement (mean difference ± 2SD). Therefore there was a good agreement between albumin and cobalt microsphere methods of measuring PSS.

In the 10 rats who underwent repeated determinations of PSS, the baseline levels were variable ranging from 17–94% with a median value of 40.5%. The reproducibility of 5 consecutive determinations of PSS in each of the animals was good with a mean coefficient of variation of 1.2%.

### DISCUSSION

It is generally accepted that there is little extrahepatic portal systemic collateral circulation in the normal rat. Therefore, the mean portal systemic shunting measurement of approximately 2.5% detected by both microsphere methods probably reflects the degree of intrahepatic portal systemic shunting. Subsequent evaluation of the degree of portal systemic shunting after partial portal vein ligation will depend on the sum of both intra-and extrahepatic shunting, although the former is negligible compared to the latter.

No significant differences were found between the two methods of determining the portal systemic shunting in sham operated rats. However, the Tc-albumin microsphere method consistently gave lower values for portal systemic shunting than the $^{57}$Co microsphere method in portal hypertensive rats. This discrepancy between the two methods of determining PSS may possibly be related to differences in the range of diameters between the $^{57}$Co and $^{99m}$ Tc microspheres used in the study. Conceivably a greater proportion of the larger albumin microspheres may be entrapped in the splenic pulp after administration. However, this explanation seems unlikely since our preliminary studies indicated that less than 2% of the injected dose of either $^{57}$Co or $^{99m}$Tc was retained in the spleen after injection. Furthermore, there was no significant difference between intrasplenic or mesenteric vein administration of either $^{57}$Co and $^{99m}$Tc microspheres. A more attractive possibility is that in the rat, a proportion of the blood vessels comprising the extrahepatic collateral circulation have a diameter less than 17 $\mu$m in which a proportion of the larger $^{99m}$Tc microspheres become entrapped. Furthermore, if the proportion of these small vessels increases as the extrahepatic collateral circulation becomes more extensive, this suggestion would also explain the greater discrepancy between the two methods with increasing degrees of PSS. Although this hypothesis could not be confirmed in the present study there was an excellent correlation between the cobalt and albumin microsphere methods. However, the relationship between two variables derived by linear regression assumes that there is no error in one of the variables. This is obviously not the case in the present study and an error exists in both methods of determining PSS. A mathematical relationship between PSS determined by $^{57}$Co and $^{99m}$Tc-albumin microsphere was therefore derived using a modification of the least square technique which minimises the sum of the squares at right angles to the curve rather than in the Y-axis only. Using this mathematical approach an equation can be derived to relate PSS determined by $^{57}$Co or $^{99m}$Tc-albumin microspheres:

$$\text{PSS shunting } (^{57}\text{Co})\% = 0.75\% \times \text{PSS shunting } (^{99m}\text{Tc})\% + 1.84\%$$

Bland and Altman[9] and Murray and Miller[10] have recently pointed out that a good correlation between the results of two methods of measuring a single parameter, in this case PSS, is not necessarily an indicator of agreement. This is borne out by the regression equations for the correlation plots of the data determined in this investigation. Testing for agreement of the method of Bland and Altman[9] indicated that there was reasonable agreement between the albumin and cobalt microsphere methods of detecting PSS. However, with increasing degrees of extrahepatic shunting the agreement between the two methods is less good. Consequently if the $^{99m}$Tc albumin microsphere method detects a 0.6% rise in shunting, the $^{57}$Co-microsphere method would detect a 1% rise. In conclusion, therefore, the quantification of portal systemic shunting by intrasplenic injection of $^{99}$Tc-albumin microspheres is comparable with the $^{57}$Co-microsphere technique. However, the $^{99}$Tc-labelled albumin microsphere method allows for repeated measurement of portal systemic shunting in experimental animals. Furthermore since the albumin microspheres are biodegradable this method could also be applied to man. Determination of collateral blood flow in portal hypertensive patients by intrasplenic injection of radiolabelled MDP and albumin microspheres could be used to measure collateral blood flow and portal pressure simultaneously. This would be particularly advantageous in patients with non-alcohol related cirrhosis with a pre-sinusoidal component to their portal hypertension since measurements of corrected wedged hepatic venous pressure (WHVP) do not accurately reflect the portal pressure. There is a risk of haemorrhage associated with splenic puncture but this can be overcome by use of fine needles and by embolising the needle track with gelfoam at the end of the procedure. Using this technique it is hoped 1) to gain further information on the role of portal pressure and collateral blood flow in the pathophysiology of variceal haemorrhage, 2) compare the relative efficacies of vasoactive drugs on portal pressure and collateral blood flow simultaneously in portal hypertensive patients and 3) determine the nature and extent of the extrahepatic shunts.

### ACKNOWLEDGEMENTS

The work described in this paper has been presented in part to the Surgical Research Society (UK), British Society of Gastroenterology and the Nuclear Medicine Society and published in abstract form in the Br. J. Surg, Gut and Nuc. Med. Commun.

### REFERENCES

1. Viallet, A., Marleau, D., Huet, M. *et al.* (1975) Haemodynamic evaluation of patients with intrahepatic portal hypertension. *Gastroenterology,* **69**, 1297–1300.
2. Reynolds, T.B. (1980) Inter-relationships of portal pressure, variceal size and upper gastrointestinal bleeding. *Gastroenterology,* **79**, 1332–1333.
3. Bosch, J. and Grossman, R.J. (1984) Measurement of azygos venous blood flow by a continuous thermal dilution technique: An index of blood flow through gastroesophageal collaterals in cirrhosis. *Hepatol,* **4**, 424–429.
4. Bosch, J., Mastai, R., Kravetz, D., *et al.* (1984) Effects of propanolol on azygos venous blood flow and hepatic and systemic haemodynamics in cirrhosis. *Hepatology,* **4**, 1200–1204.
5. Mastai, R., Bosch, J., Navasa, M., *et al.* (1986) Effects of continuous infusion and bolus injection of somatostatin (SMS) on azygos blood flow and hepatic and systemic haemodynamic in patients with portal hypertension. Comparison with vasopressin. *J. Hepatol.,* **3** (Suppl 1): 53 (Abstract).
6. Chojkier, M. and Grossman, R.J. (1981) Measurement of portal-systemic shunting in the rat by using-labelled microspheres. *Am. J. Physiol,* **240**, G371–G375.
7. Myking, A.O. and Halvorsen, J.F. (1973) A method for graded portal vein stenosis in rats: Survival related to degree of stenosis. *Eurr. Surg., Res.,* **5**, 454–457.
8. Makler, P.T. Jr. and Charles, D.M. (1980) Studies of skeletal tracer kinetics. Optimum time delay for $Tc^{99}$ ($^{m}$Sn) methylene diphosphonate bone imaging. *J. Nucl Med.,* **26**, 641–645.
9. Bland, J.M., Altman, D.G. (1986) Statistical methods for assessing agreement between two methods of clinical measurement. *Lancet,* **i**, 307–310.
10. Murry, G.D. and Miller, R. (1990) Statistical comparison of two methods of clinical measurement. *Br. J. Surg.,* **77**, 384–387.

